# Lessons learnt when accounting for competing events in the external validation of time-to-event prognostic models

**DOI:** 10.1093/ije/dyab256

**Published:** 2021-12-17

**Authors:** Chava L Ramspek, Lucy Teece, Kym I E Snell, Marie Evans, Richard D Riley, Maarten van Smeden, Nan van Geloven, Merel van Diepen

**Affiliations:** Department of Clinical Epidemiology, Leiden University Medical Center, Leiden, The Netherlands; Biostatistics Research Group, Department of Health Sciences, University of Leicester, Leicester, UK; Centre for Prognosis Research, School of Medicine, Keele University, Keele, UK; Division of Renal Medicine, Department of Clinical Science, Intervention and Technology, Karolinska Institutet and Karolinska University hospital, Stockholm, Sweden; Centre for Prognosis Research, School of Medicine, Keele University, Keele, UK; Julius Center for Health Sciences and Primary Care, University Medical Centre Utrecht, Utrecht University, Utrecht, The Netherlands; Department of Biomedical Data Sciences, Leiden University Medical Center, Leiden, The Netherlands; Department of Clinical Epidemiology, Leiden University Medical Center, Leiden, The Netherlands

**Keywords:** Prediction, prognostic model, external validation, competing risks, calibration, discrimination

## Abstract

**Background:**

External validation of prognostic models is necessary to assess the accuracy and generalizability of the model to new patients. If models are validated in a setting in which competing events occur, these competing risks should be accounted for when comparing predicted risks to observed outcomes.

**Methods:**

We discuss existing measures of calibration and discrimination that incorporate competing events for time-to-event models. These methods are illustrated using a clinical-data example concerning the prediction of kidney failure in a population with advanced chronic kidney disease (CKD), using the guideline-recommended Kidney Failure Risk Equation (KFRE). The KFRE was developed using Cox regression in a diverse population of CKD patients and has been proposed for use in patients with advanced CKD in whom death is a frequent competing event.

**Results:**

When validating the 5-year KFRE with methods that account for competing events, it becomes apparent that the 5-year KFRE considerably overestimates the real-world risk of kidney failure. The absolute overestimation was 10%age points on average and 29%age points in older high-risk patients.

**Conclusions:**

It is crucial that competing events are accounted for during external validation to provide a more reliable assessment the performance of a model in clinical settings in which competing risks occur.

Key Messages

- Competing events often occur when predicting time-to-event outcomes (apart from all-cause mortality).
- In model development, using methods with inappropriate assumptions on competing events will lead to predicted risks that are too high. This bias will not be detected in external validation studies if the validation does not explicitly account for competing risks in the statistical methods.
- Statistical methods to adapt discrimination and calibration performance measures for externally validating prognostic models in settings with competing events are available.
- The 5-year Kidney Failure Risk Equation is not suitable for risk prediction in patients with advanced kidney disease due to the overestimation of kidney failure. This overestimation can be explained completely by the competing risk of death.
- Accounting for competing risks in the statistical methods of external validation studies will provide a more reliable assessment of the performance of the model in clinical settings in which competing risks occur.

## Introduction

Prognostic models have rapidly become an integral part of medical practice. As clinical care moves towards individualized monitoring, decision-making and treatment, it is imperative to collect information on an individual’s risk profile and many prognostic models have been developed.^1–3^ External validation of prognostic models is a crucial step to assess the accuracy and generalizability of the model but may present various methodological challenges including the occurrence of competing events.^4^

Competing events prohibit patients from experiencing the prognostic outcome of interest and often occur when studying high-risk interventions, long prediction horizons or cause-specific mortality. For instance, the prediction of kidney failure in patients with advanced chronic kidney disease (CKD) is complicated due to patients dying from other causes before they can develop kidney failure. A conventional time-to-event regression model (such as a standard Cox model) that predicts an individual’s risk of kidney failure would censor all patients with incomplete follow-up in the same manner, including patients with competing events (death). Such a model would therefore overestimate the absolute risk of kidney failure.^5–7^ The overestimation of risks due to unaccounted-for competing events can result in counterintuitive and misleading prognostication. For instance, in a population with kidney failure, the 5-year risk of cardiovascular death and the 5-year risk of non-cardiovascular death sum to 107% when calculated separately without correctly accounting for competing events.^8^ The calculated probabilities are hypothetical risks assuming that no patient dies from the other cause. Though there are exceptions, ‘the risk assuming no occurrence of death’ ordinarily has little clinical relevance. In this study, we thus assume that researchers aim to estimate the absolute risk of prognostic outcomes in a real-world setting in which competing events occur. The definitions of terminology used in the current paper can be found in Box 1.

**Box 1:** Glossary

Prediction horizon	The specified time period over which predictions are made; in our clinical validation, this is 2 and 5 years
Event of interest	The primary event that is being predicted; in our clinical validation study, this is kidney failure
Competing event	Any events that may preclude the primary event from happening, in this case death without kidney failure
Absolute risk	The cumulative risk of the event of interest within the prediction horizon, given that patients may be censored and patients with a competing event will not experience the event of interest. This risk is also referred to as real-world risk, actual risk, crude risk or cumulative incidence. It can be calculated through a non-parametric cumulative incidence function, which is also termed an Aalen-Johansen estimator
Predicted risk	The risk predictions (output) from a prediction model over the specified prediction horizon. In this study, we assume that the predicted risks are available, calculated from an existing model. The accuracy and precision of these predicted risks are evaluated in external validation
Observed probability	The observed rate of the event of interest in the validation cohort, which is compared with the predicted risk. If there is no censoring and no competing events, this is the proportion of patients who experience the primary event. If competing risks and censoring are present and the researcher wants to account for this, the observed probability for a group is the same as the absolute risk (detailed above)
‘Accounting for competing events’	The use of methods that allow patients to fail from competing events. These patients are retained in the data set but dealt with using assumptions in a way that precludes them from experiencing the event of interest after the competing event, thereby differing from the assumptions for patients censored due to loss to follow-up or other reasons
‘Ignoring competing events’	Using statistical methods with inappropriate assumptions concerning competing events, most often by assuming no competing risks or that competing risks could be eliminated

The importance of using appropriate competing-risk modelling techniques (such as Fine & Gray subdistribution regression models or combined cause-specific Cox models) for prognostic model development is increasingly recognized.^6^^,^^9–16^ Nevertheless, most clinical time-to-event prognostic tools are developed using conventional regression models.^9^^,^^17^^,^^18^ Therefore, it is important to recognize that the influence of competing events can also be evaluated during external validation, as will be illustrated in this article. By doing so, existing time-to-event models can be validated in settings in which competing events may be more or less frequent than in the development setting. This paper was inspired by a recent publication from our research group in which existing kidney-failure models were validated while accounting for the competing risk of death.^19^ In this process, many lessons on involved statistics and the interpretation of results were learnt, which we hope to share.

The aim of this paper is to draw attention to the importance of externally validating time-to-event prognostic models in a manner that appropriately accounts for competing events. First, we concisely discuss the technicalities of assessing performance measures in a competing-risk setting. Second, we provide a real-data example in which we externally validate an existing prognostic model of kidney failure in patients with advanced CKD. This example illustrates the effects of competing events on measures of prognostic performance and details how such analyses can shift clinical conclusions considerably.

## Predictive performance at external validation

In this paper, we assume that the time-to-event model of interest has already been developed and may or may not have accounted for competing risks. Second, we assume that the aim is to validate this model in a setting in which competing events occur, and that clinicians and patients want individualized absolute risk predictions that reflect this. Finally, we assume a specified prediction horizon for which validation is of interest.

External validation of a prognostic model assesses the accuracy of predictions made by the model in individuals who were not used to develop the model.^20^ Important elements of prognostic model performance are assessed by comparing how well the predicted risks agree with the observed outcomes (calibration) and how well predictions separate patients who will and will not experience the outcome of interest (discrimination). We now discuss existing measures of calibration and discrimination that incorporate competing events for time-to-event models. The Supplementary Material (available as Supplementary data at *IJE* online) includes a more in-depth explanation on these various methods and we have provided a GitHub repository (in collaboration with authors from a STRATOS initiative guidance paper) with available R-code on how to validate a competing-risk model.

### Calibration

The calibration of predicted and observed outcomes can be assessed through calibration-in-the-large (overall calibration) and visualized using calibration plots.^21^ When dealing with competing events, it is key that the observed probability is calculated in a way that accurately accounts for the competing events and thereby represents the absolute risk of the event of interest.

Calibration-in-the-large can be assessed by comparing the average predicted risk for the outcome to the observed probability at the prediction horizon. Dividing the observed probability by the average predicted probability gives the O/E ratio. The average predicted risk is known, since we assume that all individual predicted risks according to the existing prediction model are given. In the case of censoring, the non-parametric Kaplan–Meier (KM) estimator is often used to calculate the observed probability. However, in the presence of competing events, the KM estimate will overestimate the absolute risk of the event of interest.^8^^,^^22^ A more appropriate method to calculate the observed outcome probability in the presence of competing events is the non-parametric cumulative incidence function (CIF).^23^ Calculating the CIF is similar to using the KM method, but quantifies the risk for the event of interest and competing events—all of which increase over time. Using the CIF, patients who experience a competing event are no longer at risk of experiencing the outcome of interest and the probability of the outcome of interest is scaled by the cumulative probability of experiencing any event. No assumptions are needed on the independence of competing events and the outcome.^6^^,^^8^

In calibration plots, the predicted and observed outcome probabilities are plotted against each other to visualize their agreement. Often the cohort is divided into subgroups based on quantiles of predicted risks. The average predicted and observed outcome probabilities for each subgroup can be computed (accounting for competing events as described above) and plotted. This approach has been criticized as the categorization is arbitrary and can lead to loss of precision and misleading results.^24^ It is therefore recommended to include a smoothed curve in the calibration plot. In the presence of censoring, this smoothed curve is often based on pseudo-values. In the presence of competing events, this smoothed curve can be obtained using pseudo-values, as described by Gerds *et al.*^25^ By using these pseudo-values that are based on cumulative incidence estimates, the model calibration is estimated over the full range of predicted probabilities.

### Discrimination

Discrimination examines the ability of the model to distinguish between those who will experience the outcome of interest from those who will not and is based on the ranked order of predicted risks.^26^ For survival data, Harrell’s C-index is the most frequently reported measure of discrimination, which is the proportion of all examinable pairs in which the individual with the highest predicted risk is observed to experience the outcome sooner than the other individual.^24^ A C-index of 1 is perfect discrimination and 0.5 is equivalent to chance. Censored patients are treated as if they might still experience the outcome in the future, which is an incorrect assumption in the case of censoring due to a competing event.^27^

In the presence of competing events, various methods to calculate a C-index are available, some of which are referenced.^11^^,^^13^^,^^28^ In the case of complete outcome data (no or very few patients are lost to follow-up), a simple adaptation of Harrell’s C-index as proposed by Wolbers *et al.* can be employed.^11^ Instead of censoring patients who experience a competing event, these patients are retained in the risk set whilst setting their follow-up time to infinity (or the prediction horizon), thus indicating that they will never experience the event of interest. In the case of only administrative censoring, also termed ‘censoring complete’, an adaptation of the Wolbers’ approach can be used in which patients with the competing event are censored at the administrative censoring date (instead of infinity).^29^^,^^30^ In the case of informative censoring, more suitable methods are available, some of which have been adapted for competing-risks settings, using inverse probability of censoring weighting (IPCW).^13^^,^^28^^,^^31^^,^^32^ In IPCW, a pseudo-population that would have been observed if no censoring occurred is created. This pseudo-population contains only patients who are followed until they experience either the event of interest, a competing event or the end of follow-up. This is done by upweighting patients who are similar to censored patients but remain in the study (under the assumptions of exchangeability, consistency and positivity). Royston and Sauerbrei’s D statistic is a measure of prognostic separation.^33^ It can be interpreted as the coefficient (log hazard ratio) for comparing two equally sized prognostic groups, created by dichotomizing the linear predictor estimates of the model in the cohort at the median value.^34^ Higher values of the D statistic represent greater separation between the survival curves for these prognostic groups. To calculate the D statistic in an external validation study, the linear predictors (for each individual) from the prognostic model are ranked and scaled. The scaled ordering of the linear predictors is then entered into a new regression model with the event of interest as the outcome; the resulting regression coefficient is the D statistic. In an external validation of a time-to-event model, the scaled linear predictor values are generally entered into a new Cox model. To adapt this measure to a setting with competing events in an external validation, the Cox model can be replaced by a Fine & Gray regression model.^35^ The D statistic can be transformed to the proportion of explained variation: *R*^2^_D_.^36^ This measure indicates how much of the observed variation in the outcome is explained by the prognostic model.

## Real-data illustration: predicting kidney failure in advanced CKD patients from the Swedish Renal Registry

### Rationale

Predicting kidney failure in advanced CKD patients is of interest for timely preparation of dialysis and transplantation, adequate monitoring of patients, possible referral back to primary care and informing patients of their likely prognosis. As the rate of progression to kidney failure highly varies between individuals, prognostic models have been proposed for use in clinical practice. The Kidney Failure Risk Equation (KFRE) is a prognostic model that was developed to predict kidney failure in patients with CKD stage 3–5 who were referred to a nephrologist.^32^ It was later externally validated and updated in a large meta-analysis and is recommended for use in international medical guidelines.^37–39^

Cox proportional-hazards models were used in the KFRE model development and external validation studies, meaning that patients who died before experiencing kidney failure were censored.^32^^,^^39–43^ This means that the predicted outcome is the risk of kidney failure in a setting in which patients are prevented from dying at least until kidney failure occurs. This risk is, however, not defined as such in the study. Instead, the predicted risk is presented as the absolute risk of kidney failure, which is more clinically relevant and conducive towards medical decision-making. In the KFRE development study, the use of a competing-risk model was explored as a sensitivity analysis but not published, as the predicted risks were deemed to be similar to those from the Cox model.

In this clinical illustration, the aim is to externally validate the KFRE in two ways, first using methods that are fitting for a Cox prediction model and treat patients with a competing event in the same way as any other censored patient (similar to the development study) and, second, using methods described previously to account for competing events in order to validate how well the KFRE predicts the real-world risk of kidney failure.

### Methods

The KFRE includes the four following predictors: age, sex, estimated glomerular filtration rate (eGFR) and urine albumin-to-creatinine ratio. The outcome of kidney failure is defined by the initiation of dialysis or kidney transplantation within 2 or 5 years. The full prediction formulae are provided in the development studies and are also shown in the Supplementary Material (available as Supplementary data at *IJE* online).

Patients were included from the Swedish Renal Registry (SRR)—an ongoing registry of CKD patients capturing 98% of the nephrology clinics in Sweden.^44^^,^^45^ Patients who entered the registry between 1 January 2012 and 30 June 2018 were included. The analysis was restricted to patients aged ≥18 years with an eGFR of between 8 and 30 ml/min/1.73 m^2^. The eGFR is a measure of kidney function; <30 indicates advanced CKD. Time zero (moment of prediction) was inclusion in the SRR, which is generally the first referral to a nephrologist.

### Results

In total, 13 489 patients were included in our analysis, of whom 1818 (13%) developed kidney failure (the outcome of interest) within 2 years and 2764 (20%) within 5 years. Slightly more patients died without experiencing kidney failure: 2158 (16%) within 2 years and 3357 (25%) within 5 years. No patients were lost to follow-up. All patients were administratively censored on 30 June 2018. The median follow-up was 1.7 years and the maximum was 6.7 years. In total, 3548 patients (26%) were administratively censored within 2 years and 6410 patients (48%) within 5 years. For each individual, the predicted 2- and 5-year risks were calculated using the KFRE formulae. Missing predictors were imputed using the R-package mice.^46^ For the illustrative purposes of this article, we used a single imputed data set for all analyses; more information on the imputation and baseline data is shown in the Supplementary Material (available as Supplementary data at *IJE* online).

**Figure 1:**
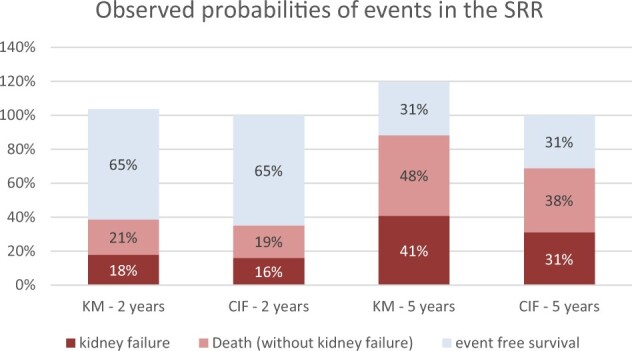
Differences between Kaplan–Meier (KM) and cumulative incidence function (CIF) estimates of the observed outcome probabilities in the presence of competing events, in the Swedish Renal Registry (SRR)

**Figure 2:**
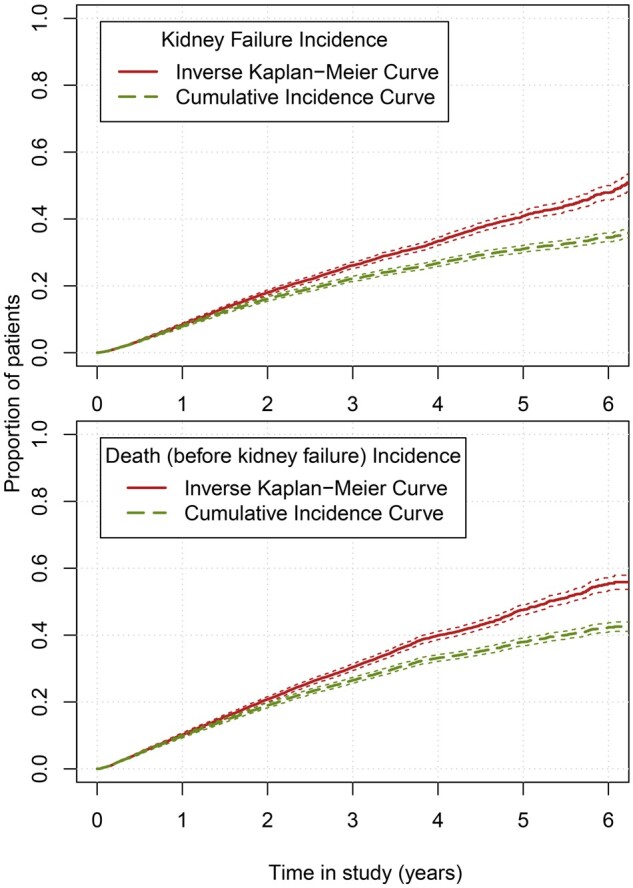
One minus Kaplan–Meier curves and cumulative incidence curves of the observed outcome probabilities in the Swedish Renal Registry for kidney failure and death. For illustrative purposes, patients who experienced kidney failure were censored or regarded as a competing event in the lower plot.

The differences between the observed outcome probabilities of kidney failure, death and event-free survival, calculated using the KM and CIF methods, are shown in a stacked histogram (Figure 1) and in cumulative incidence curves (Figure 2). At 2 years, the KM risk for death and kidney failure are both 2%age points higher than when calculated with the CIF, resulting in a total risk of 104%. At 5 years, the sum of the risks using KM increases to 120%. Risks based on the CIF method always sum to 100%.

**Figure 3:**
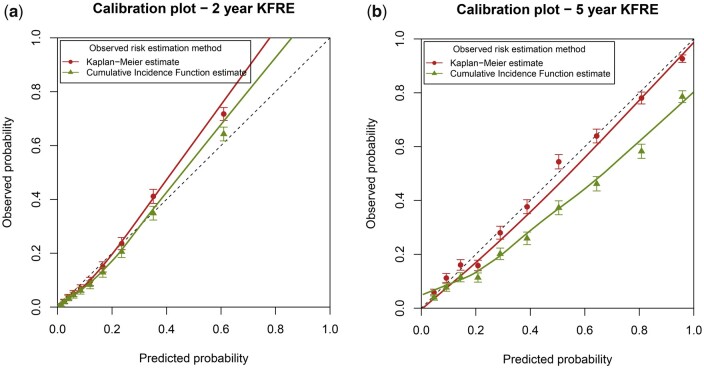
Calibration plots for external validation of the 2- and 5-year Kidney Failure Risk Equation (KFRE). The external validation was performed by using Kaplan–Meier estimates (ignoring competing risks) and by using a competing-risks approach. The competing-risks approach (green points and line) represents the model performance for the absolute kidney-failure risk in a setting in which patients may die.

To assess the calibration-in-the-large, the observed kidney-failure outcome probabilities based on KM and CIF were compared with the average predicted risk of kidney failure of the model (Table 1). The 2-year KM and 5-year KM outcome probabilities are both similar to the average predicted probability. When we consider the competing risk of death using the CIF, the observed 2-year probability of kidney failure is slightly lower but still similar to the average predicted risk of the model, with an O/E of 0.94 [95% confidence interval (CI): 0.91–0.98]. The 5-year observed probability however is almost 10%age points lower than the predicted risk, with a corresponding O/E of 0.76 (95% CI: 0.74–0.78). Similar results are seen in the calibration plot using KM and CIF. In Figure 3A, the 2-year calibration curves for both methods are quite similar. In Figure 3B, the calibration plot for the 5-year KFRE is shown. When calculating the observed probability using the standard KM method, calibration appears to be excellent. However, when we take the competing risk of death into account, the KFRE appears to considerably over-predict the actual proportion of patients with kidney failure, particularly in high-risk patients. Out of the tenth of patients with an average predicted 5-year kidney-failure risk of 81%, only 58% (95% CI: 56%–61%) experienced kidney failure.

**Table 1:** Calibration and discrimination results for external validation of the 2- and 5-year KFRE, in the entire validation cohort (*n* = 13 489). The external validation was performed in two manners, first by ignoring the competing risk of death by censoring these patients and using Kaplan–Meier estimates and second by validating the models whilst taking account of competing risks in all performance measures.

	KFRE 2-year model		KFRE 5-year model	
	Ignoring competing events by censoring	Taking competing events into account	Ignoring competing events by censoring	Taking competing events into account
Average predicted risk	17%	17%	41%	41%
Average observed probability (95% CI)	18% (17%–19%)	16% (15%–17%)	41% (40%–42%)	31% (30%–32%)
O/E ratio (95% CI)	1.06 (1.02–1.10)	0.94 (0.91–0.98)	1.00 (0.98–1.02)	0.76 (0.74–0.78)
C-index (95% CI)	0.840 (0.831–0.849)	0.834 (0.825–0.843)	0.829 (0.821–0.837)	0.814 (0.806–0.822)
D statistic (95% CI)	2.34 (2.25–2.42)	2.32 (2.20–2.43)	2.13 (2.06–2.19)	2.04 (1.95–2.14)
*R* ^2^_D_	57%	56%	52%	50%

KFRE, Kidney Failure Risk Equation; O/E, observed/expected; CI, confidence interval.

For model discrimination, the differences are less pronounced between accounting for competing risks or not (Table 1). When patients who die are censored, the standard Harrell’s C-index is 0.829 for the 5-year KFRE. When these patients are no longer censored but set to the follow-up time that they would have had if administratively censored (to indicate that patients who die will not experience kidney failure), the C-index is slightly lower: 0.814. The D statistic and explained variance also reflect that when competing risks are accounted for, the 5-year discrimination is slightly lower (Table 1).

**Figure 4:**
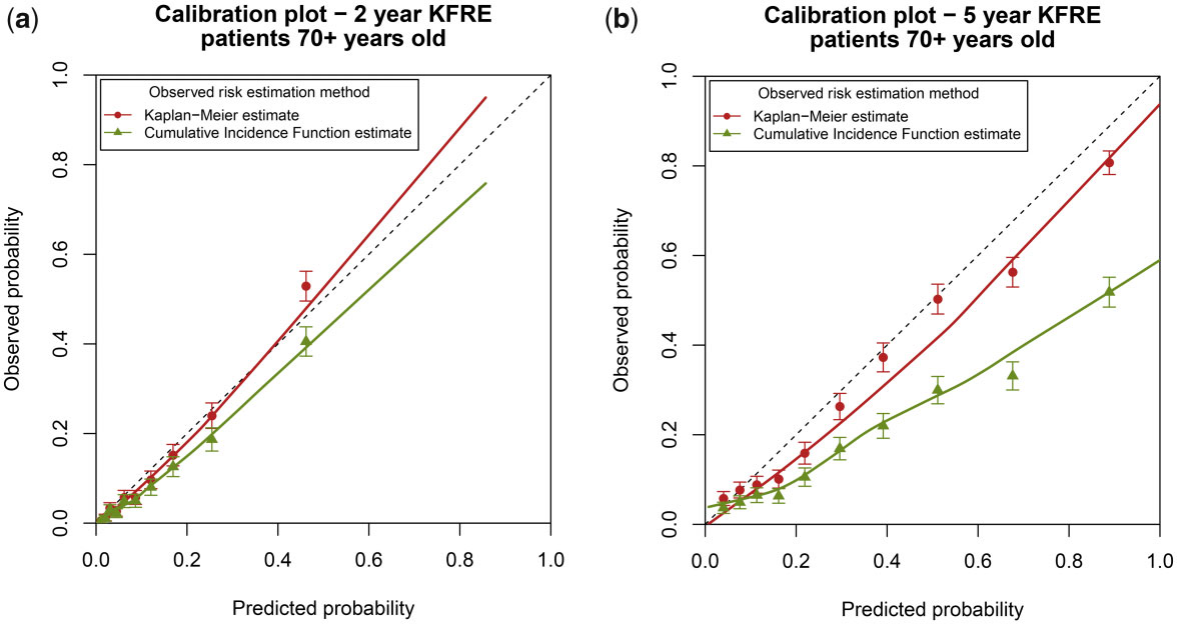
Calibration plots for external validation of the 2- and 5-year Kidney Failure Risk Equation (KFRE) in a subset of older patients. The external validation was performed by using Kaplan–Meier estimates (ignoring competing risks) and by using a competing-risks approach. The competing-risks approach (green points and line) represents the model performance for the absolute kidney-failure risk in a setting in which patients may die.

As death without kidney failure is more frequent in older CKD patients, we also validated the KFRE in a subgroup of patients who were ≥70 years old (*n* = 8654). These patients had a higher risk of death; 1064 patients (12%) experienced kidney failure within 5 years, whereas 2847 patients (33%) died without kidney failure. The median follow-up time was 1.7 years and the maximum follow-up time was 6.5 years. All analyses were repeated in this subgroup and these results are shown in Table 2 and Figure 4. Overall, the differences between ignoring competing events and accounting for them are even more pronounced in this high-risk subgroup. These differences are larger for the 5-year model and more apparent in measures of calibration than discrimination. For the 5-year model, the O/E is 0.84 (95% CI: 0.81–0.87) when ignoring competing events and 0.57 (95% CI: 0.54–0.59) when accounting for them. The 10% of patients with the highest predicted 5-year risk (right-most data point in Figure 4B) have an average predicted risk of 89%. Without considering the competing risk of death, 81% (95% CI: 78%–83%) of them are expected to experience kidney failure. However, when accounting for competing events, we observe that only 52% (95% CI: 48%–55%) of these patients actually experience kidney failure.

**Table 2:** Calibration and discrimination results for external validation of the 2- and 5-year KFRE, in a subset of patients aged ≥70 years (*n* = 8654). The external validation was performed in two manners, first by ignoring the competing risk of death by censoring these patients and using Kaplan–Meier estimates and second by validating the models whilst taking account of competing risks in all performance measures.

	KFRE 2-year model		KFRE 5-year model	
	Ignoring competing events by censoring	Taking competing events into account	Ignoring competing events by censoring	Taking competing events into account
Average predicted risk	13%	13%	34%	34%
Average observed probability (95% CI)	11% (11%–12%)	10% (9%–10%)	28% (27%–29%)	19% (18%–20%)
O/E ratio (95% CI)	0.91 (0.86–0.96)	0.78 (0.73–0.83)	0.84 (0.81–0.87)	0.57 (0.54–0.59)
C-index (95% CI)	0.826 (0.810–0.841)	0.813 (0.797–0.828)	0.817 (0.803–0.830)	0.791 (0.778–0.805)
D statistic (95% CI)	2.23 (2.10–2.36)	2.04(1.90–2.17)	2.09 (1.98–2.20)	1.75 (1.63–1.86)
*R* ^2^_D_	54.3%	49.8%	51.1%	42.1%

KFRE, Kidney Failure Risk Equation; O/E, observed/expected; CI, confidence interval.

### Conclusions

From the external validation of the KFRE in which we have taken the competing risk of death into account, we conclude that the 2-year KFRE adequately predicts the absolute risk of kidney failure in patients with advanced CKD. However, if we wish to interpret the kidney-failure risk as kidney failure in a real-world setting with competing events, the 5-year KFRE is poorly calibrated and considerably overestimates the absolute risk of kidney failure. This over-prediction is more pronounced in older patients. The difference between performance of the 2- and 5-year models can be attributed to a lower number of patients dying without kidney failure within 2 years. If clinicians interpret the 5-year KFRE estimate as the absolute kidney-failure risk (instead of the hypothetical risk given that no patient can die before kidney failure), the overestimation could lead to patients being unnecessarily prepared for dialysis (which includes vascular-access surgery and frequent hospital visits). As the four-variable 5-year KFRE substantially over-predicted kidney-failure risk when considering the competing risk of death, this model is not recommended for use in patients with advanced CKD. An alternative model that accounts for competing events, such as the 4-year Grams model, is recommended instead.^47^ This model has recently been compared head-to-head with the KFRE in an external validation study and demonstrated superior performance when accounting for the competing risk of death.^19^

## Discussion

In this paper, we highlighted the importance and implications of appropriately managing competing events during external validation. We provided explanation and tools on existing measures of calibration (O/E ratio and calibration plots) and discrimination (C-index, D statistic and *R*^2^_D_) that have been adapted to a competing-risk setting.

The importance of competing-event analyses has received increased attention in prognostic research.^6^^,^^9–11^^,^^17^^,^^18^ However, existing studies have mainly focused on the importance of using competing-risks methods in the development of prognostic models. It may well be that a prognostic model is developed in a setting with no or very few competing events and therefore a valid representation of the absolute risk for that population. However, if that model is then validated in a different population in which competing events are more frequent, it is crucial that these competing events are appropriately managed in the external validation process.

The presence of competing events may influence all model-performance measures, though in general the effect on absolute measures (calibration) is larger than on relative measures (discrimination). Researchers should carefully consider and select the risk that they wish to predict; if a model censors patients who experience a competing event, the predicted risk is the hypothetical risk in a setting in which the competing event does not exist.^48^ If death is the competing event, approaches that assume no competing risks will give a more extreme overestimation of the absolute risk in older populations and for longer prediction horizons, as shown in our data example. This overestimation will be overlooked if conventional validation methods are used.

The predicted risk of prognostic models is crucial in regard to medical decision-making. For instance, the KFRE is proposed for use in timely preparation for dialysis and kidney transplantation. Predicted risks that are too high may negatively influence clinical treatment decisions. External validation without accounting for competing risks may lead to the implementation of prognostic models that surreptitiously over-predict real-world outcomes and consequently result in overtreatment of patients.

The current study has a number of limitations. We have not developed any novel statistical approaches and do not provide information on how to adapt all available performance measures to a competing-risk setting. Particularly measures of net benefit and decision-curve analysis were outside the scope of the current paper. Additionally, further research may focus on adapted measures of the calibration slope and integrated calibration index to a setting with competing events.^49^ Furthermore, our data example is based on a single data set and some of the observed results may be attributable to sampling variability. In the future, a data-simulation study in which the outcome, competing event and censoring prevalence are varied may provide more insight into how model performance is affected in different competing-risks scenarios. Although the data example focused on the validation of the KFRE, which was developed using a Cox prognostic model, a strength of the current paper is that the discussed methods are applicable to other time-to-event models such as (flexible) parametric models, competing-risks models or machine-learning models such as random survival forests.

In conclusion, depending on the underlying clinical question, competing events may be crucial to consider when externally validating time-to-event prognostic models. If an existing prediction model has targeted the incorrect estimand, we can expect a poorer performance when validating this model while accounting for competing events (and thereby adjusting the estimand). Such external validation studies can help to determine whether such models are transportable to a real-life setting in which competing events occur.

## Ethics approval

According to Swedish law, healthcare-quality registries can be used for research. Patients have the right to opt out, but no additional individual consent is required for specific research projects. This study was approved by the Regional Ethical Review Board in Stockholm, Sweden (Dnr 2018/1591–31/2).

## Author contributions

C.L.R. and M.v.D. conceived of the study. M.E. contributed to data collection. C.L.R. wrote the initial draft of the manuscript. The analysis were performed by C.L.R. with aid from L.T. and N.v.G. All authors provided a substantial contribution to the design and writing of the study and approved the final version of the manuscript. C.L.R. is the guarantor for the study.

## Data availability

The data underlying this article cannot be shared publicly due to the privacy of individuals who participated in the study and according to Swedish law. The data can be obtained through the Swedish Renal Registry upon official request and proposal. R-code is available via a GitHub repository at https://github.com/survival-lumc/ValidationCompRisks. This repository uses different data than the current article due to data-sharing restrictions. The exact R script from our data illustration will be shared on reasonable request to the corresponding author.

## Supplementary data

Supplementary data are available at *IJE* online.

## Funding

The work on this study by M.v.D. was supported by a grant from the Dutch Kidney Foundation [grant number 16OKG12]. K.I.E.S. is funded by the National Institute for Health Research School for Primary Care Research (NIHR SPCR Launching Fellowship). The views expressed are those of the author(s) and not necessarily those of the NIHR or the Department of Health and Social Care. M.E. was funded by a grant from the Center for Innovative Medicine (CIMED) and ALF Medicin. L.T. is supported by the UK National Institute for Health Research (NIHR) Applied Research Collaboration East Midlands (ARC EM).

## Supplementary Material

dyab256_Supplementary_Data
