# Supplementary material for: Lessons learnt when accounting for competing events in the external validation of time-to-event prognostic models
Source: Int J Epidemiol. 2021 Dec 17;51(2):615–25. doi: 10.1093/ije/dyab256 (PMC9082803; doi:10.1093/ije/dyab256)
Supplement: dyab256_Supplementary_Data [file dyab256_supplementary_data.docx]

**Supplemental material for**Accounting for competing events in the external validation of time-to-event prognostic models; a practical guide and clinical example

Chava L. Ramspek^1^, Lucy Teece^2^, Kym I.E. Snell^3^, Marie Evans^4^, Richard D. Riley^3^, Maarten van Smeden^5^, Nan van Geloven^6^, Merel van Diepen^1^
^1^ Department of Clinical Epidemiology, Leiden University Medical Center, Leiden, the Netherlands.
^2^ Biostatistics Research Group, Department of Health Sciences, University of Leicester, Leicester UK.
^3^ Centre for Prognosis Research, School of Medicine, Keele University, Keele, UK.
^4^ Division of Renal Medicine, Department of Clinical Science, Intervention and Technology, Karolinska Institutet and Karolinska University hospital, Stockholm, Sweden
^5^ Julius Center for Health Sciences and Primary Care, University Medical Centre Utrecht, Utrecht University, Utrecht, Netherlands.
^6^ Department of Biomedical Data Sciences, Leiden University Medical Center, Leiden, the Netherlands

**Statistical details and code**
A GitHub repository is available at <https://github.com/survival-lumc/ValidationCompRisks>. This GitHub page accompanies a more in depth STRATOS statistical guideline on available methods to validate competing risk models from our co-author Nan van Geloven. This STRATOS statistical guideline is still in preparation at the current time of article submission. The Prediction_CSC.md in depth markdown document with script provides R-code for the validation measures discussed in our main manuscript as detailed below.

1. **Calibration-in-the-large**The O/E ratio using non-parametric cumulative incidence functions to calculate the observed probability is a measure of calibration-in-the-large or overall calibration. R-code to calculate this is shown in section 2.1.3 of the in-depth GitHub repository (in-depth markdown document). The corresponding confidence interval can be calculated according to a method by Debray which is also included in the R-code.(1)
2. **Calibration plot**

For the quantiles in a calibration plot the methods detailed under calibration-in-the-large can be used in subgroups. For a smoothed curve a non-parametric estimation method has been proposed using pseudo-observations. The R-code is provided in section 2.1.1.1 of the GitHub repository. The pseudo-observation for a particular patient is calculated by taking the weighted difference between the cumulative incidence estimate at the prediction horizon based on all patients and the same value leaving that patient out. This pseudo-observation is between 0 and 1 and functions as the observed probability for an individual patient. The advantage is that censored patients (who don’t have an event indicator) do have a pseudo-observation. After transforming the data into pseudo-observations, a smooth curve of actual risks can be obtained using a nearest-neighbor smoother. This smoother averages the pseudo-observations within a small interval using a rolling bandwidth along the observed distribution of the risk estimates.(2)

1. **C-statistic**In the case of complete outcome data, an adaptation of Harrell’s C-index as proposed by Wolbers et al. can be employed.(3) Instead of censoring patients who experience a competing event, these patients are retained in the risk set whilst setting their follow-up time to infinity (or the prediction horizon), thus indicating that they will never experience the event of interest. Pairs where one individual has the primary event (within the prediction horizon) and the other has the primary event later or experiences a competing event can be compared. The R-code is provided in section 2.2.1 of the GitHub repository. The C-index is influenced by the censoring distribution and this is particularly problematic when this censoring distribution depends heavily on other covariates.(4) When pairing cases with non-cases, Harrel’s C-index cannot evaluate a pair in which the non-case is censored at an earlier time-point than the case. These non-evaluable pairs are ignored and this may induce bias.(4) More appropriate methods for calculating the C-index in time-to-event data with independent censoring have been developed. Most of these methods use inverse probability censoring weights (IPCW). In IPCW a pseudo-population that would have been observed if each patient were a complete-case, is created. A complete-case is an individual that has either experienced the event of interest, a competing event or is still at risk at the prediction-horizon. Complete-case patients are weighed inversely to their probability of having their particular outcome. In other words, patients who were not likely to remain in follow-up (but did), are up-weighted. To minimize bias in an external validation study of a time-to-event model with a considerable number of patients with dependent censoring, we advise to use IPCW estimates of the C-index.(5)
2. **Royston-Sauerbrei D statistic and R^2^_D_**R-code for Royston and Sauerbrei’s D-statistic as measure of prognostic separation and the R^2^_D_ can be found in section 2.2.3. To calculate this, each individual’s linear predictor value is ordered and the corresponding rankits (standard normal order statistics) are calculated and scaled by a factor $k=\sqrt{8/\pi}$. The scaled rankits are regressed on the outcome using a Fine & Gray model in the case of competing events. The resulting regression coefficient is the D-statistic. The D-statistic can be scaled to the log relative hazard scale to calculate the R^2^_D_. The D-statistic and R^2^_D_ rely on a proportional hazards assumption and the assumption that the underlying linear predictor values are normally distributed (normality assumption). (6, 7)

**KFRE model**The KFRE Web calculator can be found at: <https://kidneyfailurerisk.com/>
To compute predicted risks, eGFR was calculated with the CKD-Epi formula. ACR is in mg/g, serum albumin in g/dL, phosphate in mg/dL, bicarbonate in mEq/L, calcium in mg/dL.

The following non-North America formulas were used (as provided in the KFRE eAppendix 2 of the meta-analysis and update paper).(8)
KFRE 4 variable 2-year probability = 1 – 0.9832 ^ exp (-0.2201 × (age/10 – 7.036) + 0.2467 × (male – 0.5642) – 0.5567 × (eGFR/5 – 7.222) + 0.4510 × (logACR – 5.137))

KFRE 4 variable 5-year probability = 1 – 0.9365 ^ exp (-0.2201 × (age/10 – 7.036) + 0.2467 × (male – 0.5642) – 0.5567 × (eGFR/5 – 7.222) + 0.4510 × (logACR – 5.137))

**Multiple imputation and baseline data**For the purpose of this illustration a single multiple imputation was used with 5 iterations, instead of multiple imputations. However, all suggested methods can be applied on multiply imputed data, though for calibration choices will have to be made on whether to use the predicted risk from one of the imputed datasets at random or combine predicted risks from all imputed sets for an overall mean predicted risk per individual.

ACR was the only predictor with missing values (42%). Our single imputation included the following variables as predictors at time zero to impute ACR: diabetes, hypertension, cardiovascular disease, blood pressure, albumin, calcium, phosphate, potassium, bicarbonate, eGFR, age, gender, log(ACR) at 6 months, log(ACR) at 12 months, kidney failure & time to kidney failure, death & time to death.

Baseline table of the SRR population, stratified by outcome. Continuous baseline characteristics are presented as mean values with standard deviations or median values with interquartile ranges when not normally distributed. Categorical variables are presented as valid percentages.

|  | **Missing** | **Total   n = 13489** | **No kidney failure within 5 years**  **n=10725** | **Kidney failure within 5 years n=2764** |
| --- | --- | --- | --- | --- |
| **Age (year)** | 0% | 74.3 (65.7-81.2) | 76.0 (68.5-82.2) | 66.6 (53.8-74.2) |
| **Sex (% male)** | 0% | 61.3% | 60.0% | 66.4% |
| **Primary Kidney Disease (%)** | 0% |  |  |  |
| Diabetes mellitus |  | 21.5% | 18.8% | 32.1% |
| Glomerular disease |  | 6.9% | 5.4% | 12.7% |
| Hypertension |  | 30.2% | 32.7% | 20.4% |
| Other |  | 41.4% | 43.1% | 34.8% |
| **Comorbidities (%)** |  |  |  |  |
| Congestive heart failure | 0% | 21.0% | 23.3% | 12.1% |
| Cardiovascular disease (other) | 0% | 21.3% | 23.3% | 13.5% |
| Hypertension | 0% | 73.2% | 75.0% | 66.1% |
| Diabetes mellitus | 0% | 36.4% | 35.0% | 41.9% |
| **Laboratory parameters** |  |  |  |  |
| eGFR (MDRD) (ml/min/1.73m^2^) | 0% | 21.9 (5.7) | 22.9 (5.3) | 18.2 (5.6) |
| ACR urine (mg/mmol) | 41.8% | 36 (7 - 155) | 24 (5 - 101) | 175 (57 - 340) |
| Serum Albumin (g/L) | 6.9% | 36 (5.2) | 37 (34 - 40) | 35 (32 - 39) |
| Serum Creatinine (µmol/L) | 0% | 227 (194-278) | 232 (65) | 306 (97) |
| Serum Calcium (mmol/L) | 13.4% | 2.29 (0.29) | 2.31 (0.16) | 2.24 (0.18) |
| Serum Phosphate (mmol/L) | 9.0% | 1.30 (0.29) | 1.26 (0.27) | 1.44 (0.32) |
| Serum Bicarbonate (mmol/L) | 73.4% | 22 (3.4) | 23 (3.4) | 22 (3.2) |
| Serum Potassium (mmol/L) | 54.4% | 4.43 (0.55) | 4.41 (0.53) | 4.50 (0.59) |
| **Clinical parameters** |  |  |  |  |
| Body-mass index (kg/m²) | 33.5% | 28.3 (6.0) | 28.2 (6.0) | 28.5 (6.3) |
| Systolic Blood pressure (mmHg) | 5.7% | 141 (22) | 139 (22) | 147 (22) |
| Diastolic Blood Pressure (mmHg) | 5.8% | 77 (12) | 76 (12) | 81 (13) |

Abbreviations: RRT: renal replacement therapy, eGFR: estimated Glomerular Filtration Rate, MDRD: Modification of Diet in Renal Disease formula, ACR: albumin creatinine ratio. Lab values are shown SI units and can be converted to conventional units as follows, urinary PCR in mg/g: multiply by 8.85, urinary ACR in mg/g: multiply by 8.85, serum albumin in g/dL: multiply by 0.1, serum creatinine in mg/dL: multiply by 0.011, serum calcium in mg/dL: multiply by 4.0, serum phosphate in mg/dL: multiply by 3.1.

**References**

1. Debray TP, Damen JA, Snell KI, Ensor J, Hooft L, Reitsma JB, et al. A guide to systematic review and meta-analysis of prediction model performance. Bmj. 2017;356:i6460.

2. Gerds TA, Andersen PK, Kattan MW. Calibration plots for risk prediction models in the presence of competing risks. Stat Med. 2014;33(18):3191-203.

3. Wolbers M, Koller MT, Witteman JC, Steyerberg EW. Prognostic models with competing risks: methods and application to coronary risk prediction. Epidemiology (Cambridge, Mass). 2009;20(4):555-61.

4. Uno H, Cai T, Pencina MJ, D'Agostino RB, Wei LJ. On the C-statistics for evaluating overall adequacy of risk prediction procedures with censored survival data. Stat Med. 2011;30(10):1105-17.

5. Wolbers M, Blanche P, Koller MT, Witteman JC, Gerds TA. Concordance for prognostic models with competing risks. Biostatistics. 2014;15(3):526-39.

6. Royston P, Sauerbrei W. A new measure of prognostic separation in survival data. Stat Med. 2004;23(5):723-48.

7. Teece L. Investigating the presence and impact of competing events on prognostic model research: Keele University; 2019.

8. Tangri N, Grams ME, Levey AS, Coresh J, Appel LJ, Astor BC, et al. Multinational Assessment of Accuracy of Equations for Predicting Risk of Kidney Failure: A Meta-analysis. Jama. 2016;315(2):164-74.
